# Letter to the Editor: How to raise the interest for neurogastroenterology among young gastroenterologists?

**DOI:** 10.1002/ueg2.12191

**Published:** 2022-01-03

**Authors:** Advait Upadhyaya, Manfredi d’Afflitto, Bishoy Yassa

**Affiliations:** ^1^ Wingate Institute, Barts and The London School of Medicine and Dentistry, Queen Mary University of London London UK

Dear Editor,

We found your article, ‘How to raise the interest for neurogastroenterology among young gastroenterologists?’ written by *Chloé Melchior* et al. published in the United European Gastroenterology Journal^1^ to be insightful and we wanted to reflect on a couple of the points raised and provide a medical student's perspective.

Competency‐based medical education is currently a hot topic in gastroenterology, and it's also gaining traction in neurogastroenterology and motility (NGM). Indeed, according to recent research, competency‐based training in motility should be focused on objective performance evaluations rather than the number of operations or the amount of time spent in training.^2^, ^3^ Given the high prevalence of NGM problems in children, it's crucial to have paediatric gastroenterology fellows who are well‐versed in neurogastroenterology and gastrointestinal (GI) motility disorders, as well as encouraging individual fellows and students to pursue NGM as a career path.^2^, ^3^

Despite the high prevalence and financial burden of functional GI and motility problems, only 25% of adult gastroenterology fellowship programmes include any motility training, and only 12% provide comprehensive motility training. One way to improve fellow training is to create programmes that allow for dedicated time for motility during fellowship. However, as medical students, we believe that similar schemes for undergraduates would help boost interest in the field.^3^ Early exposure to the speciality may play a key role in developing awareness and interest. Furthermore, despite differences in medical curriculum between nations, students who are placed in hospitals that provide neurogastroenterology services should be encouraged to participate in observerships in this speciality. The only hindrance to this, however, is the limited understanding and complex management of disorders of gut‐brain interaction, and hence, limited clinical application at an undergraduate level.

In conclusion, as discussed by Melchior et al., educational programmes have a great potential to increase interest in this subspeciality. Despite the importance of targeting young trainees, welcoming the participation of students in these events may provide exposure to this speciality from an earlier stage, thus potentially driving career aspirations down the line. Indeed, the prospect of organising a programme similar to Pancreas 2000, focused on neurogastroenterology, is an exciting opportunity to build an international network within the field. E‐learning programmes and prizes, along with research and clinical opportunities, may prove to be excellent drivers for the engagement of the student group.

## CONFLICT OF INTEREST

All authors declare no conflict of interest.

## Data Availability

Data sharing not applicable to this article as no datasets were generated or analysed during the current study.
